# An Asp to Strike Out Cancer? Therapeutic Possibilities Arising from Aspartate’s Emerging Roles in Cell Proliferation and Survival

**DOI:** 10.3390/biom11111666

**Published:** 2021-11-10

**Authors:** Iiro Taneli Helenius, Hanumantha Rao Madala, Jing-Ruey Joanna Yeh

**Affiliations:** 1 The Jackson Laboratory, Bar Harbor, ME 04609, USA; taneli.helenius@jax.org; 2 Cardiovascular Research Center, Massachusetts General Hospital, Charlestown, MA 02129, USA; hanupharma9@gmail.com; 3 Department of Medicine, Harvard Medical School, Boston, MA 02125, USA

**Keywords:** oxidative phosphorylation, mitochondrial respiration, mitochondrial DNA mutation, hypoxia, aspartate, glutaminase, asparagine, GOT1, alpha-ketoglutarate, cancer metabolism

## Abstract

A better understanding of the metabolic constraints of a tumor may lead to more effective anticancer treatments. Evidence has emerged in recent years shedding light on a crucial aspartate dependency of many tumor types. As a precursor for nucleotide synthesis, aspartate is indispensable for cell proliferation. Moreover, the malate–aspartate shuttle plays a key role in redox balance, and a deficit in aspartate can lead to oxidative stress. It is now recognized that aspartate biosynthesis is largely governed by mitochondrial metabolism, including respiration and glutaminolysis in cancer cells. Therefore, under conditions that suppress mitochondrial metabolism, including mutations, hypoxia, or chemical inhibitors, aspartate can become a limiting factor for tumor growth and cancer cell survival. Notably, aspartate availability has been associated with sensitivity or resistance to various therapeutics that are presently in the clinic or in clinical trials, arguing for a critical need for more effective aspartate-targeting approaches. In this review, we present current knowledge of the metabolic roles of aspartate in cancer cells and describe how cancer cells maintain aspartate levels under different metabolic states. We also highlight several promising aspartate level-modulating agents that are currently under investigation.

## 1. Introduction

Most types of cancer cells rely on both mitochondrial metabolism and glycolysis [1,2]. While a high glucose flux via glycolysis and its parallel pathway, the pentose phosphate pathway (PPP), can provide copious amounts of ATP for energy, NADPH for redox balance, and ribose-5-phosphate for nucleotide synthesis, the mitochondrial tricarboxylic acid (TCA) cycle generates other intermediates necessary for the biosynthesis of amino acids, lipids, heme, and nucleotides [1,2,3]. Therefore, to fuel the TCA cycle via glutaminolysis, many cancer cell types exhibit glutamine dependency [1,2,4]. As both glycolysis and the TCA cycle require a steady supply of electron acceptors, mitochondrial respiration (also known as oxidative phosphorylation (OXPHOS)), which recycles NADH into NAD+ via the electron transport chain (ETC), is critical for continuous nutrient oxidation in the cell [3]. Moreover, it has recently been demonstrated in two seminal papers by Birsoy et al. and Sullivan et al. that a primary role of mitochondrial respiration is to produce aspartate [5,6].

Aspartate is vital for multitudinous needs of a proliferating cell, being not only a proteinogenic amino acid, but also a major substrate for de novo synthesis of purines and pyrimidines [7]. Cellular import of aspartate appears limited to the brain and a subset of cancer cells [5,8]. Therefore, most cells can synthesize aspartate via mitochondrial glutamic-oxaloacetic transaminase 2 (GOT2), which uses the TCA cycle metabolite oxaloacetate as a substrate [5]. Aspartate production is thus fundamentally coupled to mitochondrial oxidative metabolism and respiration. Once generated in the mitochondrion, aspartate can be exported to the cytosol where it participates in myriad metabolic and biosynthetic pathways [9].

Upon being recognized by researchers for its pivotal role in cell proliferation and viability, a number of recent studies have also begun to uncover that aspartate levels in cancer cells are associated with the efficacy of various anticancer therapeutic approaches, including chemotherapy, targeted therapy, OXPHOS inhibition, and glutamine deprivation [10,11,12,13]. Moreover, encouraging preclinical results on the development of new aspartate-targeting agents are also coming to light [14,15]. Thus, a systematic review of these findings may help illuminate the path forward in the search for more effective anticancer strategies.

## 2. Roles of Aspartate in Cell Proliferation and Survival

### 2.1. Protein Synthesis, Amino Acid Metabolism, and the Urea Cycle

**Figure 1:**
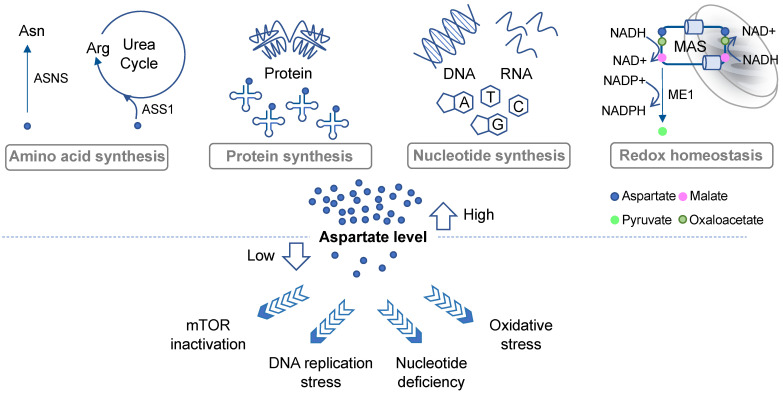
Roles of aspartate in cell proliferation and survival. The diagram outlines the major biosynthetic and redox pathways in which aspartate is required and the potential consequences when aspartate level is insufficient. ASNS, asparagine synthase; ASS1, argininosuccinate synthase 1; MAS, malate–aspartate shuttle; ME1, malic enzyme 1.

One of the important roles of aspartate in governing cell proliferation is by supporting protein and amino acid synthesis (Figure 1). Aspartate is one of the 20 proteinogenic amino acids and also serves as a precursor for the biosynthesis of asparagine and arginine. Asparate gives rise to asparagine via asparagine synthetase (ASNS). It has been shown that intracellular asparagine levels are a critical determinant of mTOR activities [16,17]. Although not all cancer cells express ASNS, such as acute lymphoblastic leukemia (ALL) blasts that mostly rely on exogenous asparagine supplies [18], in some cancer types ASNS expression has been linked to tumor progression [19,20]. In addition, aspartate is a nitrogen donor for arginine. Metabolism from aspartate to arginine is part of the urea cycle [21], whereby aspartate first reacts with citrulline via argininosuccinate synthase (ASS1) to produce argininosuccinate, which is subsequently converted into arginine and fumarate via argininosuccinate lyase (ASL). Notably, many malignancies exhibit downregulation of various urea cycle enzymes, including ASS1 [21]. ASS1 deficiency promotes cell proliferation by diverting aspartate for pyrimidine synthesis [22]. Increases in aspartate levels can also lead to activation of mTOR [22]. These results demonstrate that suppression of the urea cycle, even though it can cause arginine auxotrophy [21], provides an overall proliferation advantage to cancer cells through increased aspartate availability.

### 2.2. De Novo Nucleotide Synthesis

Proliferating cells have a high demand for nucleotides due to their robust DNA replication, RNA transcription, and ribosome biogenesis. Aspartate is a key building block for both purines and pyrimidines [7], contributing to three carbon atoms and one nitrogen atoms of the rings in uracil, cytosine, and thymine [7]. It also contributes to two purine biosynthesis steps including the conversions of 5-aminoimidazole-4-carboxamide ribonucleotide (AICAR) into succino-AICAR (SAICAR) and of inosine monophosphate (IMP) into adenylosuccinate [7]. Aspartate deprivation can result in nucleotide deficiency, DNA replication stress, and mTOR inactivation [12,14,15] (Figure 1).

In addition to cell proliferation, de novo nucleotide synthesis plays a critical role in cancer cells’ response to drugs. While studying the emergence of chemoresistant and relapse-initiating acute myeloid leukemia (AML) cells using a mouse model, van Gastel et al. found that induction chemotherapy could instigate significant metabolic changes in the stroma, resulting in its increased aspartate production and secretion [13]. Aspartate exited from the stroma could enter AML cells and increase nucleotide synthesis in these cells [13]. Consequently, inhibition of pyrimidine synthesis led to selective elimination of post-chemo relapse-initiating leukemia cells in the blood [13], suggesting that aspartate-lowering agents may in some cases reduce the advent of chemoresistance. These results further imply that investigators may need to consider the possible contribution of stroma-derived aspartate to cancer cell metabolism during the development of effective aspartate-targeting approaches.

### 2.3. Redox Balance and Oxidative Stress

Another prominent metabolic role of aspartate is to regulate cell compartmental redox balance via the malate–aspartate shuttle (MAS) (Figure 1). The net effect of the MAS is to recycle NADH produced in the cytosol (predominantly from glycolysis) back to NAD+ and to transfer the reducing power generated in the cytosol to the mitochondrion for oxidation via oxidative phosphorylation [23]. The MAS utilizes two cytosolic enzymes, glutamic-oxaloacetic transaminase 1 (GOT1) and malate dehydrogenase 1 (MDH1), to convert aspartate to malate, NADH to NAD+, and alpha-ketoglutarate (aKG; also known as 2-oxoglutarate) to glutamate. It utilizes another two enzymes in the mitochondria, GOT2 and MDH2, which in a fashion that is parallel and reverse to the cytosolic reaction, convert malate to aspartate, NAD+ to NADH, and aKG to glutamate [5]. The MAS also requires two antiporters on the mitochondrial inner membrane to transport the metabolites in and out of the mitochondria [23]. Evidence suggests that disruption of the MAS may decrease the cytosolic NAD+/NADH ratio and increase the mitochondrial NAD+/NADH ratio, which could lead to rippling effects on other metabolic pathways [9]. Moreover, some cancer types, such as those carrying *PI3KCA* mutations, appear to be more sensitive to MAS perturbation than others [24,25].

Interestingly, in KRAS-mutant pancreatic ductal adenocarcinoma (PDAC) cells, aspartate also plays an important role in maintaining redox homeostasis by supporting NADPH production [26] (Figure 1). As discussed above, aspartate is an important source for malate in the cytosol. In PDAC cells, malate is utilized by NADP-dependent malic enzyme 1 (ME1) to generate pyruvate and NADPH. Inhibition of any step in the production and utilization of cytosolic malate leads to a decrease in the NADPH/NADP+ ratio, an increase in reactive oxygen species (ROS), and a significant reduction in clonogenic growth of KRAS mutant cells [26]. Thus, once again, this study highlights the multifaceted roles of aspartate in governing cancer cell growth and survival.

## 3. Aspartate Availability, Biosynthesis, and Their Limiting Steps in Cancer

To target aspartate availability in cancer, it is important to know how intracellular aspartate level is supported. Aspartate concentration in human plasma is low, ranging between 0 and 68 µM [27]. Some cancer cells have the ability to acquire aspartate from the environment via the expression of low- or high-affinity aspartate membrane transporters, such as SLC1A2 and SLC1A3, or via macropinocytosis [8,28]. For instance, it has been shown that endocrine-resistant estrogen receptor-positive (ER+) breast cancer cells can import aspartate and glutamate, which contributes to their aggressive phenotypes and resistance to endocrine therapy [10]. For these cancer types, aspartate availability may be suppressed by targeting the expression or the activities of aspartate membrane transporters [10]. For others, it may be possible to devise an effective targeted approach by identifying the limiting steps in their respective aspartate biosynthesis pathways.

### 3.1. Aspartate Biosynthesis in Respiration-Competent Cancers

**Figure 2:**
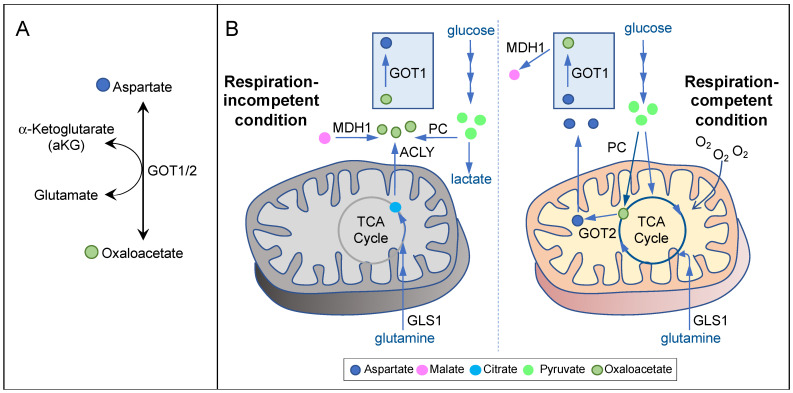
Aspartate biosynthetic pathways under different cell states. (**A**) Aspartate can be produced by glutamic-oxaloacetic transaminase (GOT) 1 or 2, which carries out the reversible reactions from oxaloacetate and glutamate to aspartate and alpha-ketoglutarate. (**B**) Aspartate biosynthetic pathways are determined by cell state, i.e., cell respiration and mitochondrial metabolism. The diagrams depict the enzymes and intermediates (shown by colored dots) involved in aspartate biosynthesis under respiration-competent (right panel) and respiration-incompetent (left panel) conditions. ACLY, ATP-citrate lyase; GOT1 or 2, glutamic-oxaloacetic transaminase 1 or 2; GLS1, glutaminase 1; MDH1, malate dehydrogenase 1; PC, pyruvate carboxylase.

In cells exhibiting mitochondrial respiration competency, aspartate is mainly synthesized in the mitochondria by GOT2 from glutamate and the TCA cycle metabolite oxaloacetate [5] (Figure 2A,B, right). Many cancer types use glutamine to fuel the TCA cycle [4,29]. Therefore, inhibition of glutaminase-1 (GLS1), the first step in glutaminolysis, in cancers displaying glutamine dependency may lead to aspartate deprivation. Indeed, while inhibition of GLS1 reduces aspartate production and the proliferation of von Hippel–Lindau (VHL)−/− renal cell carcinoma (RCC) cells, VHL+/+ cells can adapt by increasing glucose contribution to the TCA cycle and aspartate biosynthesis [12]. These results demonstrate that aspartate depletion can exert potent anticancer effects; however, it is critical that aspartate-targeting approaches should be tailored based on the specific metabolic constraints of individual cancer types.

In addition, oxaloacetate can be derived from pyruvate via pyruvate carboxylase (PC) (Figure 2B, right). A human study found that PC is overexpressed and activated in tumors of early-stage non-small cell lung cancer (NSCLC) as compared to normal lung tissues [30]. Its expression also correlates positively with advanced stages of human breast cancer samples and with breast cancer-derived lung metastases in a mouse model [31,32]. Importantly, genetic knockdown or pharmacological inhibition of PC in NSCLC and breast cancer cell lines can decrease TCA cycle metabolites, aspartate levels, cell proliferation, and the progression of xenograft tumors in mice [30,31,33]. These findings suggest that PC may be a promising target for aspartate depletion in NSCLC and late-stage breast cancer.

### 3.2. Aspartate Biosynthesis in Respiration-Incompetent Cancers

Respiration incompetency is a feature of some of the most aggressive cancer types, such as ones carrying mutations in the TCA cycle enzymes fumarate hydratase (FH) and succinate dehydrogenase (SDH) [34,35,36,37]. Tumor hypoxia or mitochondrial DNA mutations can also induce varying degrees of respiration deficiency in a wide range of cancer types [8,38]. Inhibition of mitochondrial respiration, either inducibly or inherently, can in turn cause a suppression of mitochondrial oxidative metabolism, resulting in a significant reduction in mitochondrial aspartate production [6,11]. Concordantly, aspartate is a major limiting factor for cancer cell proliferation under hypoxia or ETC inhibition both in vitro and in vivo [5,6,8,11,39].

Interestingly, it has been known for a long time that respiration-deficient cancer cells can proliferate in pyruvate-containing media [40]. Pyruvate can also confer resistance to OXPHOS inhibitors, implicating the presence of an alternative pathway for aspartate biosynthesis under respiration-incompetent conditions [11]. Indeed, Birsoy et al. showed that, in respiration-deficient cells, flux through GOT1 is reversed, so that it can generate aspartate using glutamate and oxaloacetate in the cytosol [5] (Figure 2A,B, left). In the absence of mitochondrial oxidative metabolism, oxaloacetate may be derived from three potential sources (Figure 2B, left). First, pyruvate can give rise to oxaloacetate via PC. In SDH-null cells, PC plays a pivotal role in supporting aspartate biosynthesis and cell proliferation [41]. Second, malate can give rise to oxaloacetate via MDH1 using NAD+ as the electron acceptor. Pyruvate may stimulate this reaction by recycling NADH to NAD+ via lactate dehydrogenase (LDH). In MDH1 knockout cells, pyruvate can no longer restore aspartate level in the presence of OXPHOS inhibitors [5]. Third, citrate can give rise to oxaloacetate via ATP-citrate lyase (ACLY). As citrate is generated predominantly by glutaminolysis and reductive carboxylation in respiration-impaired cells [42,43,44], glutamine utilization may also play a role in maintaining aspartate level in these cells.

Notably, essentially all of the metabolic routes described above require GOT1 for generating aspartate under respiration incompetency. While GOT1-null cells can proliferate under respiration-competent conditions, they cannot survive OXPHOS inhibition even with pyruvate supplementation [5]. These findings strongly suggest that GOT1 may be a prime target against cancers exhibiting respiration incompetency.

## 4. Therapeutic Approaches Targeting Aspartate Availability in Cancer

Various therapeutic approaches discussed herein are summarized in Table 1.

### 4.1. Glutaminase Inhibition Reduces Glutamine Utilization and Aspartate Synthesis

**Figure 3:**
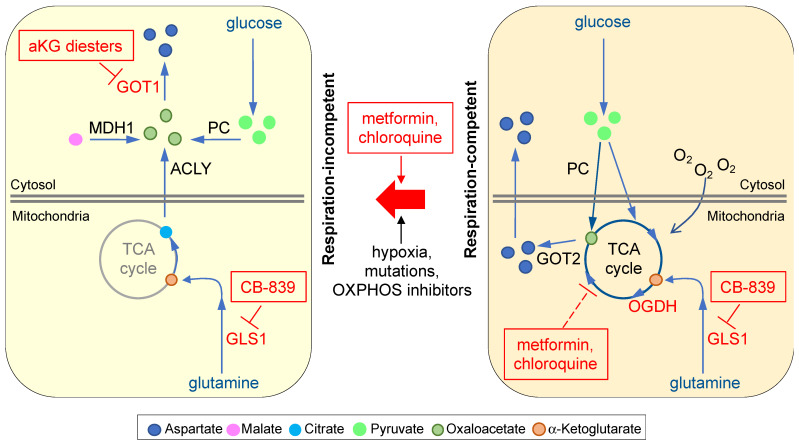
Therapeutic opportunities for abrogating aspartate availability. Protein targets (in red letters, unboxed) and chemical agents (in red letters, boxed) that have already been demonstrated to deplete aspartate in cancer cells or mouse models are shown. Metformin and chloroquine suppress the TCA cycle indirectly. aKG, alpha-ketoglutarate; ACLY, ATP-citrate lyase; GOT1 or 2, glutamic-oxaloacetic transaminase 1 or 2; GLS1, glutaminase 1; MDH1, malate dehydrogenase 1; OGDH, oxoglutarate dehydrogenase; PC, pyruvate carboxylase.

Glutamine is the most abundant amino acid in human plasma [4] and enters cells via a number of cell membrane transporters [47]. Many canonical oncogenes, including MYC and KRAS, elicit glutamine dependency [4]. Upregulation of glutamine metabolism may also contribute to anticancer treatment resistance [48,49]. Thus, pharmacological inhibition of GLS1 is an area under intensive investigation [50]. CB-839 is presently the most advanced GLS1 inhibitor in clinical development with more than a dozen Phase II combination trials underway (https://www.clinicaltrials.gov, accessed on 17 October 2021). CB-839 significantly abates aspartate in VHL-mutated RCC cells and chemoresistant ovarian cancer cells, consistent with the role of glutamine as the major fuel for the TCA cycle in these cells [12,49]. One of these studies further shows that aspartate depletion leads to nucleotide deficiency and DNA replication stress, consequently sensitizing CB-839-treated cells to poly (ADP-ribose) polymerase (PARP) inhibition [12]. These results demonstrate that aspartate depletion is an important mechanism by which glutaminase inhibitors exert anticancer effects (Figure 3). They further imply that cancers may develop resistance to GLS1 inhibitors if they can use other means to boost aspartate availability. Indeed, p53 can contribute to cancer cell adaptation to glutamine deprivation by promoting the expression of the aspartate transporter SLC1A3 [51]. Nonetheless, pathways that are particularly important for cell survival when aspartate level is low, such as PARP-mediated DNA repair, remain an attractive combination therapy target in conjunction with glutaminase inhibitors.

### 4.2. OXPHOS Inhibition Suppresses Mitochondrial Metabolism and Aspartate Synthesis

OXPHOS inhibitors are promising anticancer therapeutics because they have been shown to suppress the growth of a wide spectrum of cancer cell lines in culture and in preclinical mouse models [52,53]. In addition, some cancer types, such as RB1-deficient breast cancers, KRAS-driven cancers, and chemoresistant melanoma, are particularly reliant on OXPHOS activity and are especially sensitive to OXPHOS inhibition [54,55,56]. Thus, there are nearly a dozen OXPHOS inhibitors being investigated in the clinic and more being developed in laboratories for cancer treatment [52]. Among them, the antidiabetic drug metformin is the most studied, with over 100 ongoing clinical trials [57]. Epidemiological and clinical studies suggest that metformin decreases cancer risk when given in the absence of a cancer diagnosis and increases the survival rate of cancer patients [1,52,57]. While normalization of blood glucose and insulin levels may partially account for metformin’s anticancer effects, it is clear that metformin can directly suppress tumor growth via ETC complex I inhibition [58]. Although OXPHOS deficiency can lead to bioenergetic catastrophe in glucose-limited conditions [59], data from cell culture and mouse xenograft tumor models strongly suggest that, in normal glucose conditions, aspartate availability is a major determinant of cancer cell susceptibility to metformin and other OXPHOS inhibitors [5,6,8,11,39] (Figure 3). Therefore, a number of factors likely influence the therapeutic efficacy of OXPHOS inhibitors, including cellular import of aspartate and alternative metabolic pathways that maintain NAD+ level and thereby support aspartate biosynthesis under respiration deficiency. It is also possible that some cancer cells, under aspartate limitation, restrict aspartate utilization by certain metabolic pathways in order to conserve aspartate to prioritize supporting nucleotide synthesis [21]. Therefore, in the clinic, OXPHOS inhibition alone may not be sufficiently effective in most cancer types. It is important to note that the aspartate level of a cell is the key determinant of whether it will proliferate, enter cell cycle arrest, or die in response to compromised ETC activity [5]. Thus, as further discussed below, investigation of additional strategies to robustly reduce aspartate levels in combination with OXPHOS inhibitors are expected to generate more broadly applicable anticancer approaches.

### 4.3. aKG Depletes Aspartate via GOT1 under Respiration-Incompetent Cancer Conditions

Respiration incompetency plays a natural and critical role in the tumorigenesis of many malignancies, including cancers caused by FH and SDH mutations [34,35,36], cancers harboring pathogenic mitochondrial DNA mutations [38], or cancers residing in hypoxic environments [8]. In these cancer conditions, respiration deficiency can lead to metabolic rewiring in which GOT1 becomes the main aspartate producer [5] (Figure 2A,B, left). By examining a collection of snap-frozen human glioblastoma samples, Garcia-Bermudez et al. found that hypoxic tumors exhibit significantly reduced aspartate levels [8]. Moreover, several studies have shown that cells harboring mutations in *FH*, *SDH*, or the ETC complex III *cytochrome b* gene (*CYTB*) possess much lower aspartate levels compared to otherwise isogenic cells that do not carry these mutations [14,41]. Considering that respiration-incompetent cancer types are typically aggressive and refractory to standard anticancer therapies, their metabolic constraints in aspartate biosynthesis may represent a new therapeutic opportunity. 

Using multiple respiration-competent and -incompetent cancer cell pairs, Madala et al. identified that, at low millimolar concentrations, cell-permeable alpha-ketoglutarate (aKG) diesters induce potent cell death selectively in respiration-incompetent cells [14] (Figure 3). The proposed mechanism resulting in the specificity of this class of compounds is intriguing. The study shows that treatment with dimethyl alpha-ketoglutarate (dmaKG) leads to a surge of intracellular aKG, a key TCA cycle intermediate. While aKG accumulation subsides rapidly due to mitochondrial oxidative metabolism under respiration-competent cancer conditions, aKG might not be metabolized as efficiently under respiration incompetency. Consequently, a high concentration of aKG in the cytosol of respiration-incompetent cells reverses the flux of GOT1, switching the role of GOT1 from synthesis to depletion of aspartate in these cells [14]. The action of dmaKG is unique and different from other agents that target GOT. In contrast to treatment with the GOT1/2 inhibitor aminooxyacetic acid (AOA), which decreases aspartate level and suppresses cell proliferation indiscriminately between respiration-competent and -incompetent cells, dmaKG exhausts aspartate to a cytotoxic level selectively in respiration-incompetent cells [14]. In preclinical mouse models, dmaKG displays a promising biosafety profile as well as encouraging anticancer efficacy and specificity [14]. In light of these findings, future clinical investigations of dmaKG as a targeted therapy for various respiration-incompetent cancer types and conditions are warranted. 

Interestingly, dmaKG can also synergize with OXPHOS inhibitors to induce cytotoxicity in a wide range of respiration-competent cancer cell types [14,45]. A 2-week combined treatment with dmaKG and phenformin (a membrane-permeable analog of metformin) abolishes tumor progression and suppresses aspartate levels in xenograft tumors, but not in mouse organs, revealing a potential therapeutic window for this combination therapy [14]. As some emerging clinical evidence suggests that OXPHOS inhibition alone may not provide as significant a therapeutic benefit as originally hoped [45], further exploration of dmaKG and OXPHOS inhibitor combination therapy may prove useful. 

### 4.4. Knockdown of Oxoglutarate Dehydrogenase (OGDH) Reduces Aspartate Level in Subsets of Cancers

OGDH is the E1 subunit of the mitochondrial alpha-ketoglutarate dehydrogenase complex (KGDHC), which catalyzes oxidative decarboxylation of aKG. Two groups have independently identified different subsets of cancer cell lines that are highly sensitive to OGDH knockdown, including those harboring PIK3CA mutations [24,25]. Importantly, both studies show that OGDH knockdown results in a strong reduction of aspartate level accompanied by a decrease in the NAD+/NADH ratio in the sensitive cell types (Figure 3). The authors conclude that OGDH-dependent cancer cell lines demonstrate a profound reliance on the malate-aspartate shuttle, which makes them vulnerable to aspartate deprivation [24,25]. Interestingly, Ilic et al. showed that PIK3CA mutant cancers exhibit dmaKG sensitivity, suggesting that elevation of aKG level upon OGDH knockdown may be the cause of aspartate depletion in these cells [25]. Notably, treatment with two known chemical inhibitors of KGDHC, succinyl phosphonate (SP) and CPI-613, did not exert similar effects to OGDH knockdown [24]. Thus, a pharmacological approach targeting OGDH dependency in these subsets of cancers is still lacking. Nonetheless, the results from Ilic et al. suggest that OGDH-dependent cancer types may also be susceptible to dmaKG treatment [25]. 

### 4.5. Lysosomal Inhibition by Chloroquine Causes Aspartate Depletion

Anticancer effects of the antimalarial drug chloroquine (CQ) have been reported in multiple cancer types [60]. CQ suppresses cancer cell growth by inhibiting nutrient acquisition and recycling processes, such as autophagy and macropinocytosis, via lysosomal deacidification [60]. Surprisingly, two groups have concordantly shown that, in pancreatic ductal adenocarcinoma (PDAC) and hepatoblastoma, treatment with CQ led to a significant reduction of aspartate levels, whereas the other amino acids were largely unaffected [15,46]. In cell culture, aspartate supplementation reversed CQ-mediated DNA replication stress and cell death, suggesting that aspartate deprivation plays a crucial role in CQ’s anticancer effects [15,46]. Overexpression of SLC1A3 also reversed CQ’s effect in a xenograft tumor mouse model [15]. The mechanism by which CQ reduces aspartate level is presently unknown, although some evidence suggests that mitochondrial metabolism might be inhibited in CQ-treated cells [15] (Figure 3). Thus, it will be interesting to see whether these findings can be extended to other CQ-sensitive cancer types, and to further understand CQ’s mode of action in aspartate depletion. While clear anticancer effects of CQ have been observed in experimental models, thus far its clinical efficacy has been limited [61]. Results from Elliott et al. using a mouse model suggest that inhibition of the replication stress response (RSR) pathway by VE-822, an inhibitor of ataxia telangiectasia and Rad3-related protein (ATR) that is currently under clinical investigations, may improve the therapeutic efficacy of CQ [15].

## 5. Conclusions

Cancer cells exhibit a range of distinct metabolic phenotypes and metabolic constraints, which in principle could be leveraged for therapeutic benefit by starving cancer cells of essential nutrients. Development and evaluation of metabolically-targeted anticancer agents, however, has revealed that in many cases response to therapy is less robust than expected, both in animal models and in the clinic. One contributing factor is metabolic plasticity, whereby cancer cells can engage alternative pathways to meet their specific metabolic needs. Even so, some constraints may be insurmountable due to inflexibility imposed by genetic or environmental factors. Here, we have described the multiple critical roles of aspartate that have nominated aspartate uptake and biosynthesis pathways as potential therapeutic targets in cancer. Notably, several different anticancer agents that were originally designed with a different therapeutic rationale and target in mind in fact lead to depletion of aspartate. Cancer cells that can acquire or synthesize aspartate by compensatory means may have a particular adaptive survival advantage. Thus, co-targeting aspartate availability could enhance therapeutic efficacy, including of OXPHOS inhibitors. These concepts have now been shown in various preclinical models, and the time is ripe to move the most promising combinatorial approaches towards the clinic.

## Figures and Tables

**Table 1:** Therapeutic approaches that deplete aspartate in cancer cells.

Therapeutic Agent	Proposed Mechanism of Action	Status of Development	Co-Targeting Strategy	References
CB-839	Inhibit GLS1	clinical trial	Inhibit poly(ADP-ribose) polymerase (PARP)	[12]
Metformin	Inhibit Complex I of the electron transport chain	FDA-approved diabetes drug	Inhibit GOT1 or exploit GOT1 to exhaust aspartate	[6,11,14]
Dimethyl alpha-ketoglutarate (dmaKG)	Engage GOT1 in aspartate consumption	Pre-clinical testing	Inhibit mitochondrial respiration	[14,45]
Chloroquine	Unclear; possibly through inhibition of mitochondrial metabolism	FDA-approved anti-malarial drug	Inhibit replication stress response	[15,46]
	OGDH inhibition			[24,25]
